# The last will: Estate divisions as a testament of to whom altruism is directed

**DOI:** 10.1371/journal.pone.0254492

**Published:** 2021-07-28

**Authors:** Mikael Elinder, Per Engström, Oscar Erixson

**Affiliations:** 1 Department of Economics, Uppsala University, Uppsala, Sweden; 2 The Research Institute of Industrial Economics, Stockholm, Sweden; 3 Institute of Housing and Urban Research, Uppsala University, Uppsala, Sweden; Middlesex University, UNITED KINGDOM

## Abstract

We use data on estate divisions to study to whom altruistic preferences are directed. Insofar bequests are given without the prospect of future personal benefits in mind, they are presumably intrinsically motivated. Hence, estate divisions provide a rare opportunity to study intrinsically motivated prosocial behavior in the field. The empirical analysis is based on data from digitized estate reports for all individuals in Sweden who passed away in 2002 and 2003. The data show in detail how the decedents distributed their bequests. We find that family members, both genetic (offspring) and non-genetic (partner), receive the lion’s share of the estates. Other relatives, friends and strangers (represented by charities) receive only very small shares of the total estate wealth. The results suggest that intrinsically motivated altruism is primarily directed towards close family members.

## I. Introduction

Humans frequently engage in prosocial behavior by helping family members, friends and strangers or by contributing to charitable causes. But who are we willing to help when the possibility of receiving future reciprocal favors is excluded; in other words, who do we really care about? We analyze to whom altruistic preferences are directed by studying exceptionally rich data on how decedents chose to distribute their estate wealth between different groups of heirs (e.g., children, relatives, friends and charities).

There are almost no real-life situations, outside the lab, where the possibility of reputational motives for prosocial behavior can confidently be ruled out. Since an estate division represents the last will of a decedent, it can be seen as the last move in a repeated game of social interactions. Hence, estate divisions provide a rare real-life situation in which the influence of reputational motives is minimized and intrinsic motives for prosocial behavior can be studied.

In the model presented by Bénabou and Tirole [1], motives for prosocial behavior are classified into three broad groups: intrinsic, extrinsic and reputational. This framework encompasses several important theories on altruism and prosocial behavior. Reputational motives reflect expectations of non-contractual reciprocal benefits from the beneficiaries of an action or by third parties, as in reciprocal altruism [2], indirect reciprocity [3] and costly signaling [4]. Extrinsic motives reflect standard economic incentives, such as explicit monetary rewards, whereas intrinsic motivation corresponds to a genuine willingness to act in another person’s interest, even if doing so is costly, and corresponds well with influential definitions of altruism [5, 6]. Intrinsically motivated prosocial behavior is a key element of kin altruism [7], pure altruism [8] and strong reciprocity [9, 10]. Warm glow giving [8, 11] and giving due to fairness concerns [12] may also be interpreted as based on intrinsically motivated prosocial behavior. In the following, we use the term altruism for intrinsically motivated prosocial behavior.

From both a theoretical and a policy perspective, it is important to understand behavioral motives, as policies aiming to increase prosocial behavior may be ineffective or backfire if the underlying motives are poorly understood [13, 14]. A key prediction in the model by Bénabou and Tirole [1] is that monetary incentives may crowd out reputational motives but not intrinsic motives. Furthermore, if charitable giving is motivated by intrinsic rather than reputational motives, that would mean that highlighting the act of giving will not increase contributions. The motives for prosocial behavior may, however, vary depending on the relationship with the beneficiary. Better knowledge regarding whether prosocial behavior is driven by either intrinsic or reputational motives is thus valuable for efficient policy design.

Economic theory and policy recommendations have predominantly relied on the assumption that agents are exclusively motivated by self-interest. While charitable giving [15, 16], resource allocation within families [16] and behavior in disasters [17, 18] provide some notable exceptions, there is a lack of knowledge concerning the interactions in which altruism plays an important role and when it may be ignored. The most common approach in empirical studies on altruism has been to observe outcomes in lab experiments. However, as pointed out by Fehr and Schmidt [6], it is important to learn more about the extent to which altruism and other prosocial behaviors are conditioned on the identity or characteristics of the potential beneficiaries. In other words, who the relevant reference agents are.

Theoretical guidance on who the relevant reference agents are is provided by models in sociobiology predicting that altruism increases with genetic closeness [7] and is stronger towards cooperators than non-cooperators [9, 10]. Evidence of altruism towards cooperators has been found in lab experiments such as trust games [10, 19, 20]. Furthermore, there is ample lab evidence of the other side of this coin (i.e., costly punishment of non-cooperators), see, for instance, [10, 21–24].

The evidence on how the relationship between the giver and potential recipients affects altruism almost exclusively originates from lab and survey experiments using variations of the dictator game [25–30]. These findings suggest that altruism is strong towards kin [28, 29] and beneficiaries of charities [26] but also substantial towards anonymous strangers [26–29] as well as known non-kin [25, 29]. One persistent finding related to this is the so-called *identifiable victim effect*, which refers to the tendency that people are more prone to help identifiable victims. This phenomenon was originally alluded to by Schelling [31], and lab experiments suggest that the effect is particularly pronounced when a single victim is identified, as opposed to a group of victims [32].

Some studies [33, 34] have also shown that generosity in dictator games decreases dramatically when the subjects have to share earned money, as opposed to windfall gains.

All studies discussed above have offered invaluable insights regarding altruistic behavior. At the same time, they have also been limited by small samples, narrow sets of potential recipients and small or no stakes. Giving in the lab has also been found to be sensitive to variations in framing [35–38]. Furthermore, some scholars have also questioned whether reputational motives can be ruled out in lab experiments [26, 39] and there is a discussion on to what extent the results from lab experiments may be generalized to the field [35, 39, 40].

In contrast, an estate division is a real-life situation where substantial resources are divided between an unlimited set of potential recipients. The question arises, however, as to why individuals leave bequests. A large theoretical literature presents various explanations. Bequests may be accidental [41], stem from altruism towards heirs [16, 42] or serve as reciprocal payments for services received from, for instance, children having provided care for the parent, as in the exchange model of bequests [43, 44]. However, the empirical evidence provides limited guidance as to which model best explains bequest behavior [45]. While the models explicitly assuming altruism towards children [16, 42] are clearly aligned with the biological literature on kin-based altruism, the exchange model requires a comment. In this model, altruism is not explicitly assumed. However, individuals need to credibly commit to bequeath to those who provide them with services. This commitment problem is a key aspect of the model but can be solved by assuming that the bequeather has a genuine concern for the service providers. As stated by Bernheim et al. [43, page 160]: “presumably […] all transfers are made to individuals (or institutions) about whom the benefactor cares very much.” The type of altruism solving the commitment problem is analogous to the concept of strong reciprocity and the behavior of cooperators in trust games. In the exchange model, the decedent honors his or her word and reciprocates, by means of a bequest, those who have been cooperative, even though such a commitment cannot be enforced.

We argue that estate divisions provide unique opportunities for eliciting to whom altruistic preferences are directed. First, although no setting can rule out behavior motivated by concerns for postmortem reputation (including beliefs regarding the afterlife and reputational consequences for, for instance, the surviving family), we can rule out future interaction with the heirs and third parties. After the final estate division, social interactions between the decedent and his or her heirs cannot take place. Hence, estate divisions can be thought of as the final iteration of a game. This makes the bequest setting unique, not only in comparison to other real-life situations but also compared to the lab setting. Even in lab experiments, it is essentially impossible to rule out future interactions when the set of recipients contains family members or friends (e.g., fellow students).

Second, the estate division concerns a significant amount of money (on average $40,000 in our data) to be divided among a set of relevant recipients. Furthermore, the massive opposition to estate taxation observed worldwide suggests that individuals are concerned about what happens to their estate [46]. How the estate is to be divided is also a decision that the individual has typically had much time to carefully contemplate. Taken together, these aspects suggest that an estate division is likely to reflect the preferences of the decedent. One concern, however, is that the “nudge” provided by the default succession rules makes estate divisions less useful in terms of characterizing the preferences of the decedents (see, for instance, [47–50] for evidence on how default options influence behavior). As a robustness check, we thus show that the qualitative results are unaffected by dropping all deceased individuals who did not communicate their last will through a testament.

Third, estate divisions do not impose any restrictions on the set of potential recipients. Anyone can be a beneficiary of a bequest, including family members, friends, community members or even strangers or specific animals, as represented by charities.

The few studies having analyzed bequests to charities have shown that typically only 3–6 percent of decedents have included a charity in their testament [51], suggesting that altruism towards family members and relatives is stronger than altruism towards non-kin. However, charitable bequests are more common among the very wealthy [52, 53]. The study most closely related to ours is Smith et al. [54], who analyzed a sample of 1,000 probated testaments from Vancouver, Canada, to test the predictions from Hamilton’s rule [7]–that altruism should increase in genetic relatedness–and found that estate divisions are consistent with kin-based altruism. We extend their framework in two important ways. First, we relate the findings to several influential theories on altruism. Second, we use much richer data, thereby allowing us to present more detailed and precise estimates regarding to whom altruism is directed. Based on the results in [54] and Hamilton’s rule, our main hypothesis is that close family members receive more than other relatives, who, in turn, receive more than non-relatives, who receive more than charities.

## II. Methods and data

Below, we first present an analytical framework, which we use to formalize our hypotheses and provide a theoretical reference for our interpretation of the empirical results. Second, we present the estimation method. Finally, we discuss the details of the data.

### Analytical framework

An estate division can be interpreted as a simple and general game, which we refer to as the bequest game. The bequest game consists of two types of players: the giver (the decedent) and the recipients (the heirs). The giver has an endowment, the estate, which has to be distributed among a set of potential recipients. The giver is free to divide the endowment in whichever way he or she chooses. When the distribution decision is made, the giver passes away and the endowment is divided according to this decision.

This game is similar to the dictator game, or the last move in a trust game, in that one player alone determines the outcomes for the other players. Whether this game is more analogous to a dictator game or a trust game only has limited implications for how the results are interpreted. The exchange motive for bequests lends support to the trust game interpretation, which makes the altruism involved conditional on favors received. However, a bequest is still a sign of altruism towards the recipient insofar that future interactions can be ruled out–only the strong reciprocators reciprocate in a trust game [9, 10]. In contrast to the dictator game, the giver’s choice is not between dividing the endowment between himself/herself and another player but between a set of other players. The fact that the giver dies guarantees that future interactions with the recipients or third parties are not possible.

In order to interpret our results in terms of parameters of a utility function, we assume that the giver acts in order to maximize a standard log-linear warm glow utility function [8], in which the utility of the giver depends on the payoffs of the recipients. These payoffs, in turn, depend on the bequest (*B*_*ij*_) given to each recipient *i* belonging to recipient group *j*. We are primarily interested in these four groups: close family (*cf*), other relatives (*or*), non-relatives (*nr*) and charities (*ch*). If we denote the number of potential recipients in each group by *n*_*j*_, we can write the utility function of a representative giver as,

$$U(B)=\sum_{j=cf}^{j=ch}\sum_{i=1}^{i=n_{j}}\alpha_{ij}lnB_{ij}$$ (1)

A convenient feature of the log-linear utility function is that utility-maximizing bequests will be directly proportional to the corresponding *α*_*ij*_. Furthermore, when normalizing so that $\sum_{j=cf}^{j=ch}\sum_{i=1}^{i=n_{j}}\alpha_{ij}\equiv 1$, the optimal share of the total estate given to recipient *i* belonging to group *j* will simply be *α*_*ij*_. This means that the utility parameters *α*_*ij*_ are directly estimated by the shares received by the respective recipients.

Due to the open-ended nature of the recipient groups, in particular non-relatives and charities, it is not empirically feasible to estimate each individual *α*_*ij*_. Instead, we define $\alpha_{j}\equiv \sum_{i=1}^{i=n_{j}}\alpha_{ij}$ so that *α*_*j*_ is the aggregate relative altruism directed towards all members of group *j*. It then becomes straightforward to estimate the elements of the *α*-vector (*α* ≡ {*α*_*cf*_, *α*_*or*_, *α*_*nr*_, *α*_*ch*_}) with *α*_*j*_ as the average share of the estate, which is given to all recipients in a specific group.

The ***α***_***j***_ coefficients should be interpreted as the relative strength of altruism directed towards the respective recipient group. We cannot estimate the absolute strength of altruism or rule out spite (e.g., negative reciprocity) as a motive for not bequeathing to someone or not leaving bequests at all. Therefore, our interpretation of relative altruism assumes that all decedents are at least somewhat altruistic. While we abstract from the motive for leaving a bequest, positive estates are typically viewed as a result of a bequest motive arising from dynastic preferences [42] or stemming from precautionary savings [41, 55], so-called accidental bequests. However, neither of these two approaches rules out the possibility that the donor has preferences in terms of how the subsequent estate should be divided.

From the described framework, we derive our main hypothesis, which follows from Hamilton’s rule [7] and previous evidence from estate divisions [54]. Our main hypothesis to be tested is that the following ranking will be observed:

$$\alpha_{cf}>\alpha_{or}>\alpha_{nr}>\alpha_{ch}.$$

In other words, our hypothesis states that close family members receive more than other relatives, who, in turn, receive more than non-relatives, who receive more than charities.

### Estimation method

The objective of the estimation procedure is to produce estimates of the population/sample mean shares of the estate, which are then transferred to each of the four recipient groups (close family (*cf*), other relatives (*or*), non-relatives (*nr*) and charity (*ch*)) together with a 99 percent confidence interval.

We begin by expanding the data so that each decedent (*i*) appears four times (i.e., one per recipient group). We construct indicator variables $D_{i}^{j}$ for each recipient group (*j* = cf, or, nr, ch), which take the value one (= 1) for the specific group and otherwise zero (= 0). The outcome variable *y*_*ij*_ is the share of the decedent’s (*i*) estate going to recipient group (*j*).

To account for the fact that each decedent contributes fourfold to the estimation of the coefficients, we apply a mixed effects model. The regression model can be specified as follows:

$$y_{ij}=\alpha_{cf}D_{i}^{cf}+\alpha_{or}D_{i}^{or}+\alpha_{nr}D_{i}^{nr}+\alpha_{ch}D_{i}^{ch}+U_{i}+\varepsilon_{ij},$$ (2)

where *α*_*cf*_, *α*_*or*_, *α*_*nr*_, *α*_*ch*_ are regression coefficients, *U*_*i*_ captures the random component and *ε*_*ij*_ is the error term. We estimate the model using the statistical software Stata (version 16).

### Data and population

Our analyses are based on digitized estate reports for all individuals in Sweden who passed away (excluding minors below the age of 18) during 2002 and 2003 with an estate of positive value (146,657 individuals) and all their heirs (460,034 individuals). In accordance with Swedish law, no consent has been requested from individuals included in the registers or this study. The data have been analyzed anonymously (the personal identity numbers of the study subjects have been de-identified by Statistics Sweden). We use information concerning decedent-heir relationships, inheritances and estates for calculating the share of the estate given to four all-encompassing and mutually exclusive groups of recipients: close family (offspring and partner; i.e., spouse, registered partner or cohabiter), other relatives (parents, siblings, siblings’ offspring, grandparents, aunts and uncles), non-relatives (e.g., friends and other acquaintances) and charities.

In Sweden, as in many other countries, inheritance legislation stipulates who should inherit if the decedent does not have a testament. The last will can thus be expressed in two ways: either explicitly through a formal testament or implicitly by accepting the default division rules. A testament can be rewritten or canceled at any time and its content is typically not revealed explicitly before the demise (public announcements of testament content/intent, such as the Giving Pledge, are extremely rare in Sweden). Our data show that 24 percent of the decedents had expressed their last will in a testament. For the other 76 percent, who did not have a testament, it is more difficult to know whether they consciously chose not to write a testament since their preferences were in line with the default rules or whether they were unconsciously affected by the default rules. However, the Swedish Fundraising Association has shown that the majority of Swedes without a testament explicitly state a desire to divide the estate according to the intestate default (see S1 Appendix, section S1, for details about this survey). This suggests that estate divisions represent conscious decisions. Furthermore, when the decision maker has ample time to consider the consequences of a choice, the decision is more likely to be rational as opposed to influenced by behavioral biases [56]. We thus maintain the assumption that the final estate division represents the last will of all decedents. Nevertheless, we report results for the subset of decedents who did have a testament in S1 Appendix, section S2. As it turns out, estates are divided very similarly by decedents with and without a testament, supporting our assumption that the final estate division represents the last will also among decedents without a testament. Details about the Swedish inheritance laws are provided in S1 Appendix, section S3.

Table 1 presents descriptive statistics for the sample decedents, who were on average 80 years old when they passed away, consisted of more women than men and passed away with an average estate worth SEK 317,000 (approx. USD 40,000). The estate size is highly skewed with the median being only about half of the mean (SEK 165,000) and only 5 percent of the estates amounting to more than SEK 1 million. The estate is valued according to principles for inheritance taxation, see Elinder, Erixson and Waldenström [53] for details regarding how different assets are valued.

**Table 1:** Descriptive statistics for the decedents.

Variable	Mean	Min	Max	SD
Age (years)	80.5	18	103	11.7
Females (%)	51.9			
Estate size	317	1	473,077	1,456
Number of heirs	3.1	1	61	2.9
N	146,657			

Note: Estate values are reported in thousands of SEK at the 2003 price level. The exchange rates as of December 30, 2003: 7.8 SEK/USD and 9.0 SEK/EUR.

S1 Appendix contains further details about the data. See S1 Appendix, section S4 for details on estate divisions, S5 for further details on the grouping of recipients, S6 for the construction of samples, S7 for details on the measurement of estates and inheritances, S8 for further descriptive statistics (including statistics for decedents with zero estate wealth and those with a testament) and S9 for instructions on how to access the data.

## III. Results

Our main hypothesis states that close family members receive more than other relatives, who, in turn, receive more than non-relatives, who receive more than charities. We test this hypothesis by first estimating how the estate wealth is distributed in the full population of decedents. The estimated shares received by each group corresponds to the *α-*coefficients described in the analytical framework (Section II). However, since not all decedents have children or a partner, we proceed, in a second analysis, by re-estimating the distribution for decedents with at least one close family member. In a third analysis, we focus on decedents without a close family but with other relatives. This analysis allows us to focus on the ranking between other relatives, non-relatives and charities. In a fourth analysis, we analyze the small group of decedents without any relatives, which allows us to focus on the relative preferences between non-relatives and charities. Finally, in an additional analysis, we try to separate out whether offspring are preferred to partners.

**Fig 1:**
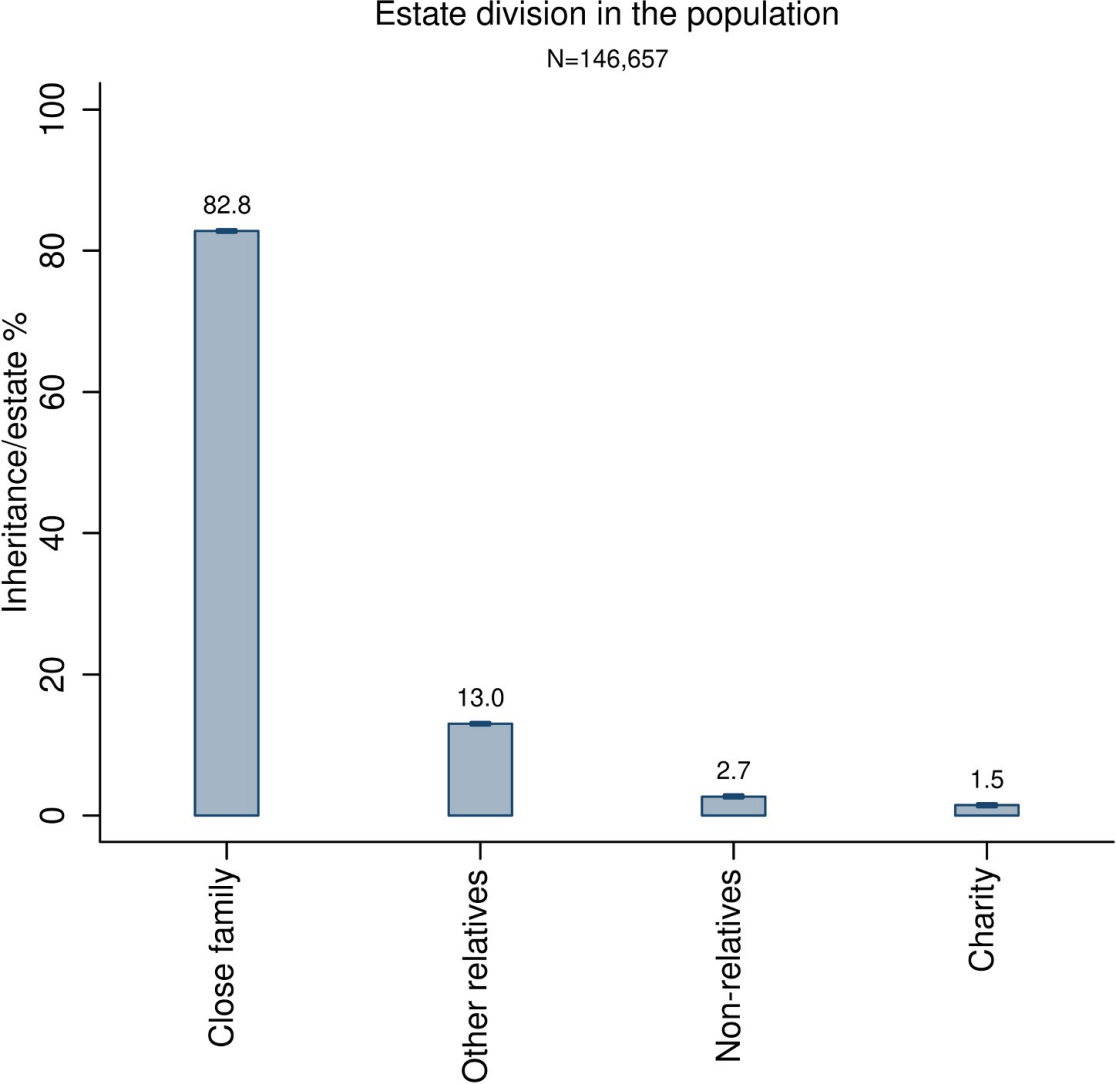
Division of estates in the full study population. The bars are accompanied by 99% confidence intervals.

Fig 1 reports the estimates for all decedents. It shows that the lion’s share of the estate (82.8%) is given to close family members. A smaller share is given to other relatives (13.0%) and very small shares are given to non-relatives (2.7%) and charities (1.5%). In S1 Appendix, section S10, we also report estimates of relative altruism towards different categories of charities.

**Fig 2:**
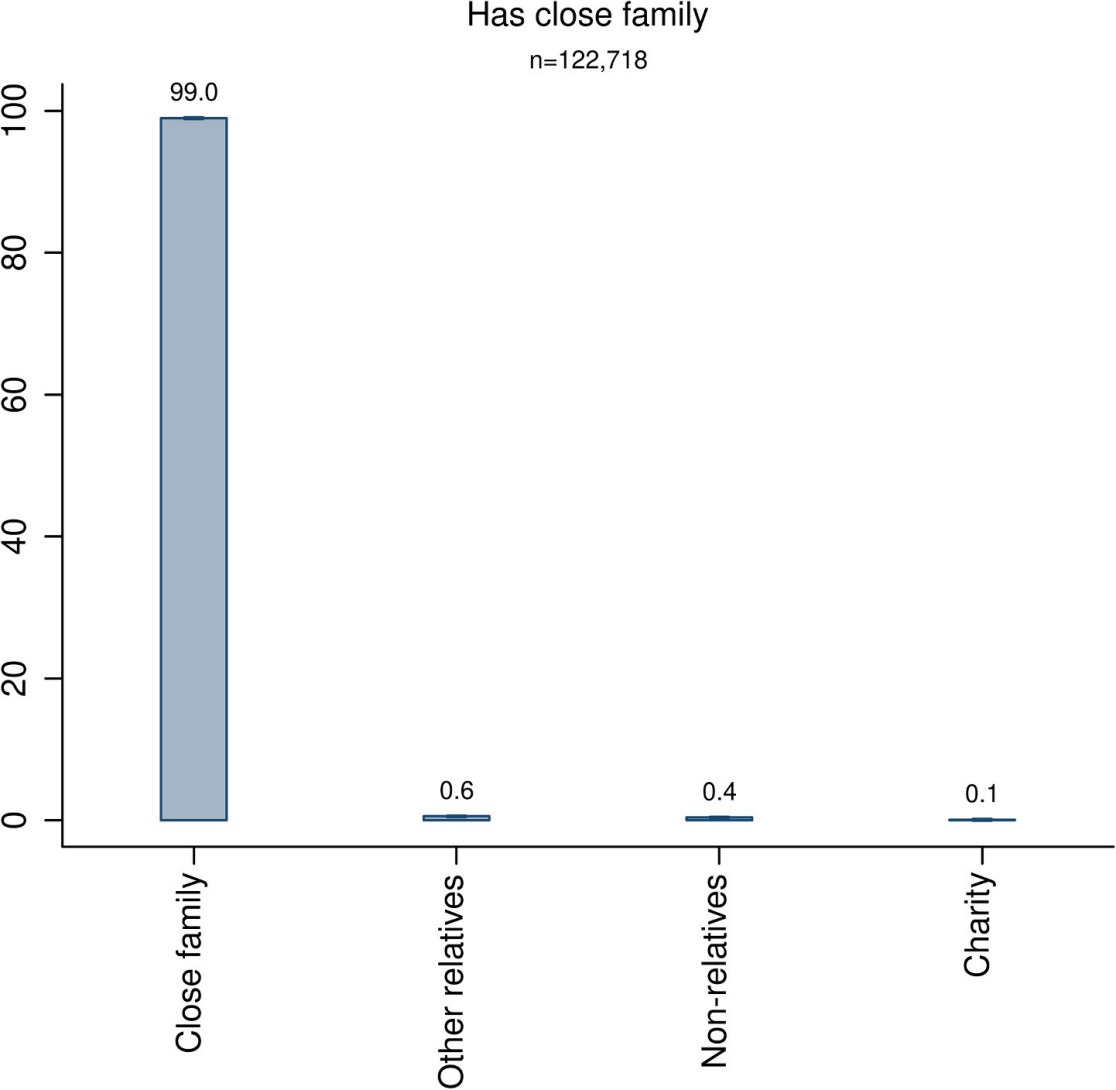
Division of estates by decedents with close family. The bars are accompanied by 99% confidence intervals.

While almost all decedents had relatives (98%) and everyone was able to bequeath to non-relatives and charities, 16.3 percent of the decedents did not have any close family member. We thus estimate preference weights for decedents who were able to bequeath to all groups of heirs. Fig 2 shows the results and reveals that for decedents with at least one close family member, almost all of the estate (99%) is given to the close family. From a policy perspective, however, it is valuable to know how decedents who do bequeath to charities differ from the average decedent, and the data show one notable feature being that they are substantially wealthier than the average decedent (see S1 Appendix, section S8).

**Fig 3:**
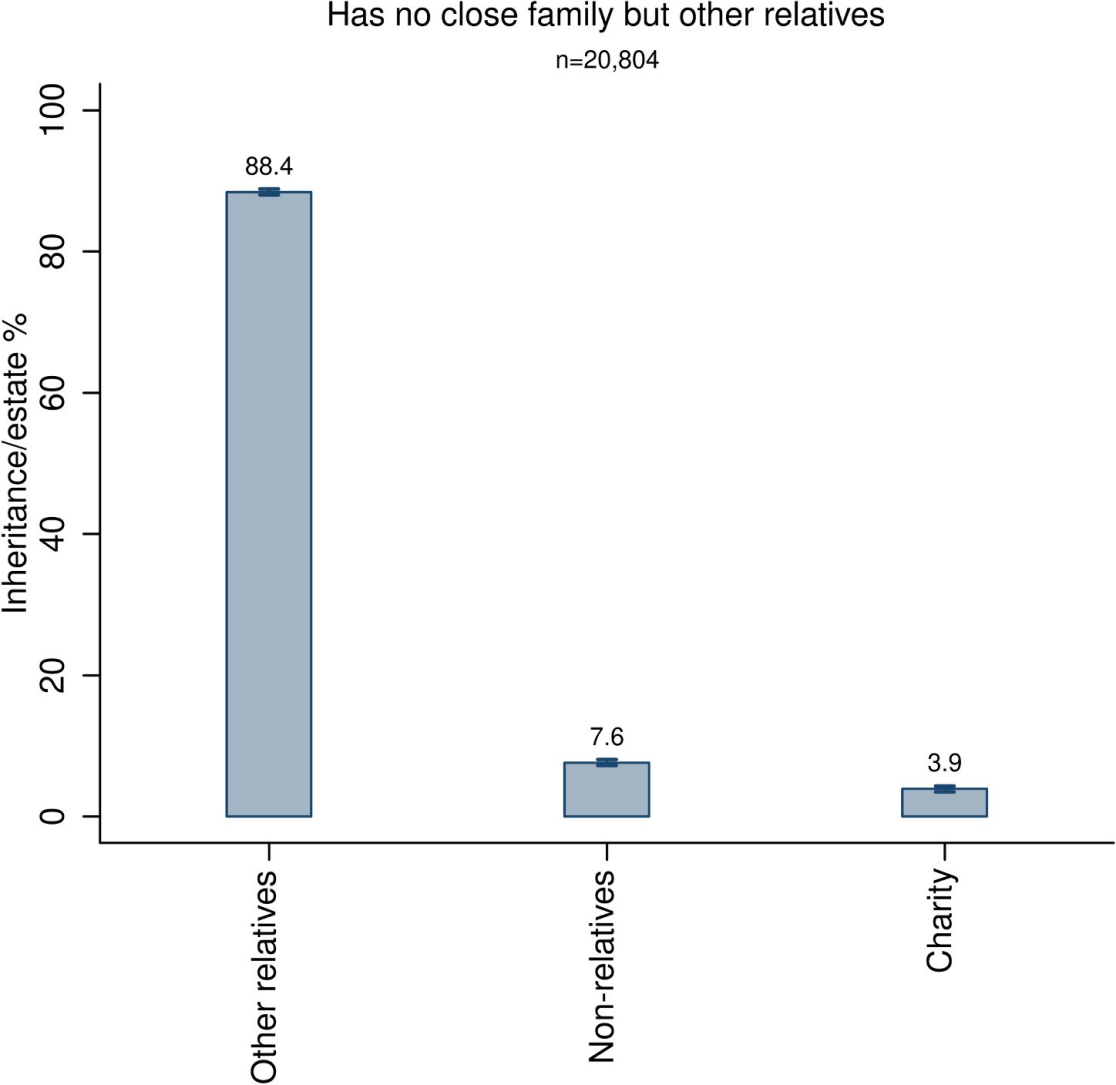
Division of estates by decedents without close family but with other relatives. The bars are accompanied by 99% confidence intervals.

In order to more accurately estimate the relative altruism parameters between other relatives, non-relatives and charities, we conduct a third test where we analyze estate divisions among decedents without close family members but with at least one other relative. Fig 3 presents the results. The vast majority of the estate (88.4%) is given to other relatives, while 7.6 percent and 3.9 percent are given to non-relatives and charities respectively. The preference ordering between these groups of recipients is the same as the ones in Figs 1 and 2. However, the relative strength of the altruism directed towards the different groups differs dramatically. Decedents without close family give about ten times more to other relatives than to non-relatives, while decedents with close family (Fig 2) give approximately the same share to other relatives as to non-relatives.

**Fig 4:**
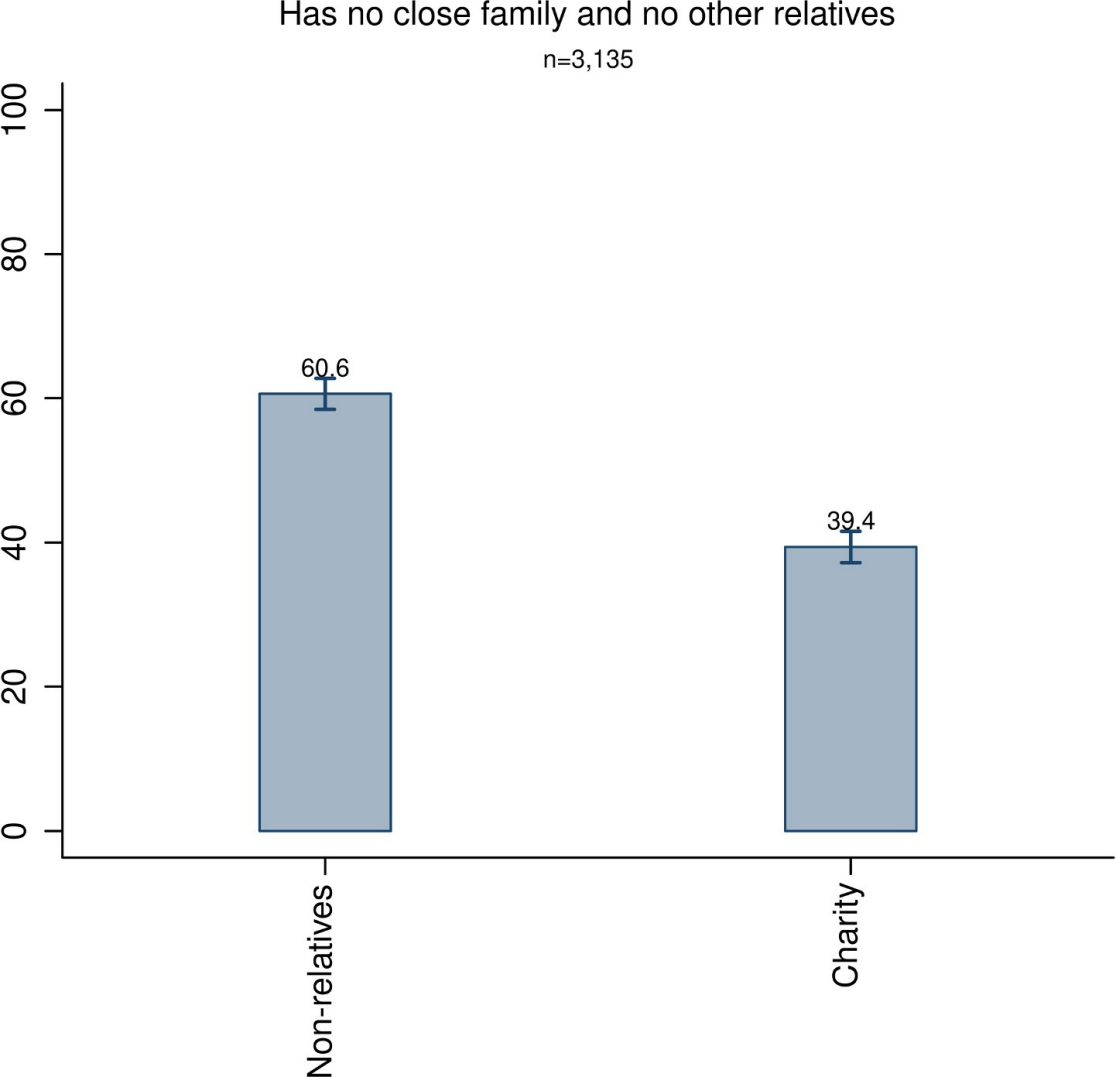
Division of estates by decedents without close family or other relatives. The bars are accompanied by 99% confidence intervals.

Finally, we go one step further and analyze the group of decedents who did not have any close family or any other relatives. Fig 4 presents the results and shows that the majority of the estate (60.6%) is given to non-relatives but also that a significant fraction is given to charity (39.4%). This group of decedents is on average wealthier than the average decedent (see S1 Appendix, section S8), indicating that kin-based altruism is not the sole reason for leaving bequests. Although these decedents only constitute 2.1 percent of all decedents, they contribute with half (50.3%) of the total amount of charitable bequests.

**Fig 5:**
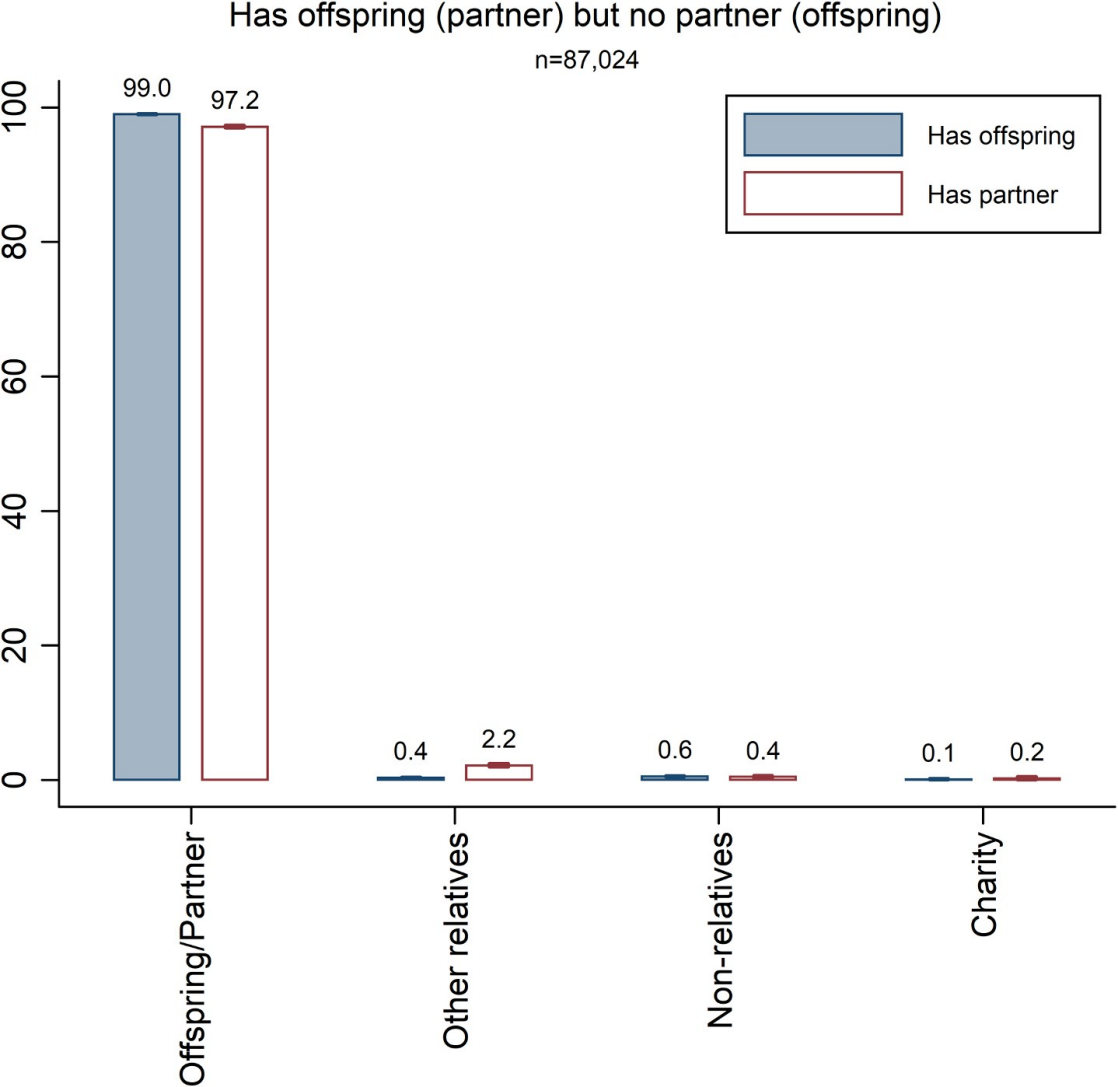
Division of estates by decedents with close family. The bars are accompanied by 99% confidence intervals.

So far, both partners and offspring are included in the group of close family. However, offspring are genetically related to the decedent, while the partner is not. We would thus expect decedents to be more generous towards offspring than to partners, as partners can only indirectly increase the survival of the decedent’s genes [7]. According to the inheritance legislation, a bequest to a surviving spouse is by default transferred to the offspring when the spouse eventually passes away. This means that it is not possible to assess whether altruism is stronger towards genetically related family members (offspring) or non-genetic family members (partners) on the basis of a comparison of how much is given to offspring relative to partners. Instead, we compare estimates for decedents *with a partner but without offspring* with estimates for decedents *with offspring but without a partner* to assess whether altruism towards offspring is stronger than that towards partners. Our hypothesis for this test is that decedents bequeath more to offspring than to partners. Fig 5 presents the results and shows that altruism towards offspring appears to be only marginally stronger than that towards partners (99.0% vs. 97.2%). While the two estimates are statistically different (p<0.01), the difference is very small. The results also reveal that partners receive much more than any other recipient group. Decedents with a partner but without offspring only give 2.2 percent of the estate to genetically related heirs. Detailed estimation results are reported in S1 Appendix, section S11.

## IV. Discussion

The results presented in Figs 1–5 suggest that altruism is predominantly directed towards genetic and non-genetic members of the close family and to a much lesser degree towards any other beneficiaries. These results are consistent with our main hypothesis that altruism is stronger towards one’s close family compared to the other groups and that altruism towards relatives is stronger than that towards friends and strangers, represented by non-relatives and charities. The results are also consistent with the main giving patterns in Smith et al. [54].

The result that individuals able to bequeath to all groups give almost all of their estate to close family members suggests that altruism towards close family is much stronger than that towards all other potential recipients.

How much stronger? Based on our analytical framework assuming log-linear utility and warm glow altruism, the relative altruism is directly proportional to the amount given to a specific group. We find that a representative decedent with close family gives 99 percent of his or her estate to the close family and only 0.1 percent to charity. The implied interpretation is that the decedent is about 1,000 times more altruistic towards his or her close family compared to all potential recipients of charitable contributions combined.

The observation that decedents without close family bequeath most of their estate to other relatives can be interpreted as if other relatives serve as substitutes for a close family. The result that partners receive more than genetic relatives (other than offspring) is inconsistent with kin-based altruism. From a kin-based altruism perspective, genetic relatives should trump non-relatives, while individuals without offspring should favor other relatives before partners. It is possible that, from an evolutionary perspective, altruism towards partners constitutes a broadly successful heuristic that misfires when two partners do not have common offspring [57]. However, partners can be viewed as important cooperators, which means that bequests to partners are consistent with strong reciprocity [9, 10].

Our results add to the policy discussion on how to promote prosocial behavior (see, for example, [38, 58]). For instance, while prosocial behavior towards family members appears to be intrinsically motivated, charitable giving during life may perhaps instead be predominantly motivated by reputational concerns [26, 59]. Policies such as making testament content visible, such as in the Giving Pledge, may thus increase charitable bequests. Moreover, organizations asking individuals to include a charity in their testament are more likely to be successful when targeting individuals without children or partners.

A few comments on the interpretation of our results are warranted.

First, a limitation in our study is that we cannot know whether the altruism displayed in the last will is representative of preferences in other stages of life. If bequests primarily represent payments for services received from the designated heirs, then the estate distribution may just mirror the balance of outstanding debts at death, rather than deeper preference parameters. It is difficult to assess the importance of this concern. However, the fact that every heir had a unique relationship with the decedent but that children almost always receive equal shares of the estate (see, for example, [60–62]) suggests that estate divisions are not only repayments for outstanding debts.

Second, social norms may influence estate divisions in two ways. If they are internalized, they have become part of one’s preferences and the individual would like to follow such norms even without social pressure to do so [63]. But, if they are not internalized, social norms can be viewed as a restriction from the individual’s perspective. In our setting, parents may feel pressure to bequeath to children. However, the presence of such a norm does not imply that bequeathing a small share of the estate to a charity would be frowned upon. On the contrary, many individuals appear to perceive it as virtuous to include a charity in the testament, as documented by Sanders and Smith [64]. Our interpretation is thus that the limited amounts bequeathed to charities are not due to social norms prescribing that all of the estate should be transferred to the children. There is, however, evidence of strong social norms prescribing that all children should inherit equal amounts (see, for example, [62]).

Third, since we present the first results, representative of an entire population, on how estates are divided between different groups of recipients, we cannot directly evaluate how well our results generalize to other countries. However, the fact that the lion’s share of estates also appear to be bequeathed to the close family in the US [61] and that the share making a charitable bequest in Sweden (3.2%) is comparable to estimates (3–6%) for five other countries [51] suggests that our results are informative for other contexts as well.

Finally, this context presents natural limitations for making conclusions regarding altruism. For instance, our results concern generosity with monetary resources. It remains to be studied whether generosity with, for instance, time presents a similar or different picture of altruism.

Our results suggest that altruism towards strangers, represented by charities, is relatively weak for all groups except individuals without any relatives. This finding complements the findings from, for example, dictator games in which substantial generosity towards strangers is commonly observed. However, our setting is quite different from the dictator game in several dimensions. Importantly, estates generally consist of hard-earned money as compared to money provided by the experimenter. The fact that generosity towards strangers is lower when the money to be distributed has been earned rather than given to the allocator, the so-called entitlement effect, has been well-established in dictator games (see, for example, [34, 35]). In this dimension, our context thus more resembles the setting in these latter experiments with earned money.

## Supporting information

S1 Appendix Supporting information. (DOCX)

S2 Appendix Novus data. (XLSX)
