# Supplementary material for: The last will: Estate divisions as a testament of to whom altruism is directed
Source: PLoS One. 2021 Jul 28;16(7):e0254492. doi: 10.1371/journal.pone.0254492 (PMC8318293; doi:10.1371/journal.pone.0254492)
Supplement: S1 Appendix — (DOCX) [file pone.0254492.s001.docx]

S1 Appendix - Supporting information

# S1 Survey Evidence

In this section, we describe the analyses underlying the claims that social norms create pressure to bequeath to charity and that a majority of Swedes without a testament want to divide the estate according to the intestate default.

The data we use for these analyses are collected from a survey conducted by Novus, one of Sweden’s largest survey companies, on behalf of the Swedish Fundraising Council (FRII), which is a membership organization for 150 fundraising organizations in Sweden. The purpose of the survey is to elicit information regarding attitudes towards testament writing and charitable bequests. The survey has been conducted in a nationally representative web panel consisting of individuals 30 years and older in 2017, and the number of respondents is 1,027 individuals (response rate 62%).

The claim that “the majority of Swedes without a testament have an explicit desire to divide the estate according to the intestate default” is based on the observation that of the 321 respondents answering that they “will probably not write a testament,” 84% answer that this is either because “everything should go to the children/family” or “it is not necessary—the inheritance rules are appropriate” (translation by the authors).

# S2 Estate divisions among decedents with testaments

To assess whether the observed estate divisions are driven by the default option or by a genuine desire, we compare the results presented in Figure 1 with the corresponding estate divisions by decedents with a testament. Decedents with a testament are likely to have made a conscious decision on how the estate should be divided. However, testament writing is not randomly distributed in the population. Rather, individuals displeased with the default succession rules are likely to be overrepresented among testament-writers. If the results were to be interpreted as representative of the population, the altruism weight given to family members would thus be underestimated. However, testaments do not necessarily alter the default division of the estate. Some may stipulate how particular assets should be divided, without altering the values of the bequests.

Figure S2 reproduces Figure 1 for decedents with a written testament. Looking first at panel A (Population), we see that the share transferred to close family falls in comparison to the corresponding share in Figure 1, while the shares to the other categories increase in relative terms. This pattern is repeated in panels B–D, implying that a smaller share is given to the default category from decedents who have consciously decided on the distribution of their estates. However, the preference ordering of the recipient categories remains similar to that presented in panels B–D in Figure 1 and close family still dwarfs all other recipient categories. This result supports our conclusion that the main estimates in Figure 1 reflect genuine preferences and are not substantially distorted by the default option. In panel E, we see that the share going to non-relatives increases while the share transferred to charity decreases in comparison to the corresponding share in panel E in Figure 1. The reason for this is that fewer inheritances are transferred to the Swedish Inheritance Fund, which is the default recipient in the absence of legal heirs and beneficiaries of a testament. In panel F, we report how the share going to charity in panel A is distributed among the various groups of charities described in S5. The pattern should be compared to the one in Figure S10. Now, the largest share goes to *Research,* in line with what has been found in the United Kingdom [52], instead of the *Swedish Inheritance* *Fund* (*SIF*). That a smaller share is now going to SIF is expected given that the primary reason for writing a testament when SIF is the default heir is to consciously set aside the default succession order. That a positive share still goes to SIF may, for instance, be explained by statements in the testament that a particular amount or share of the estate, but not the entire estate, should be given to someone else than SIF.


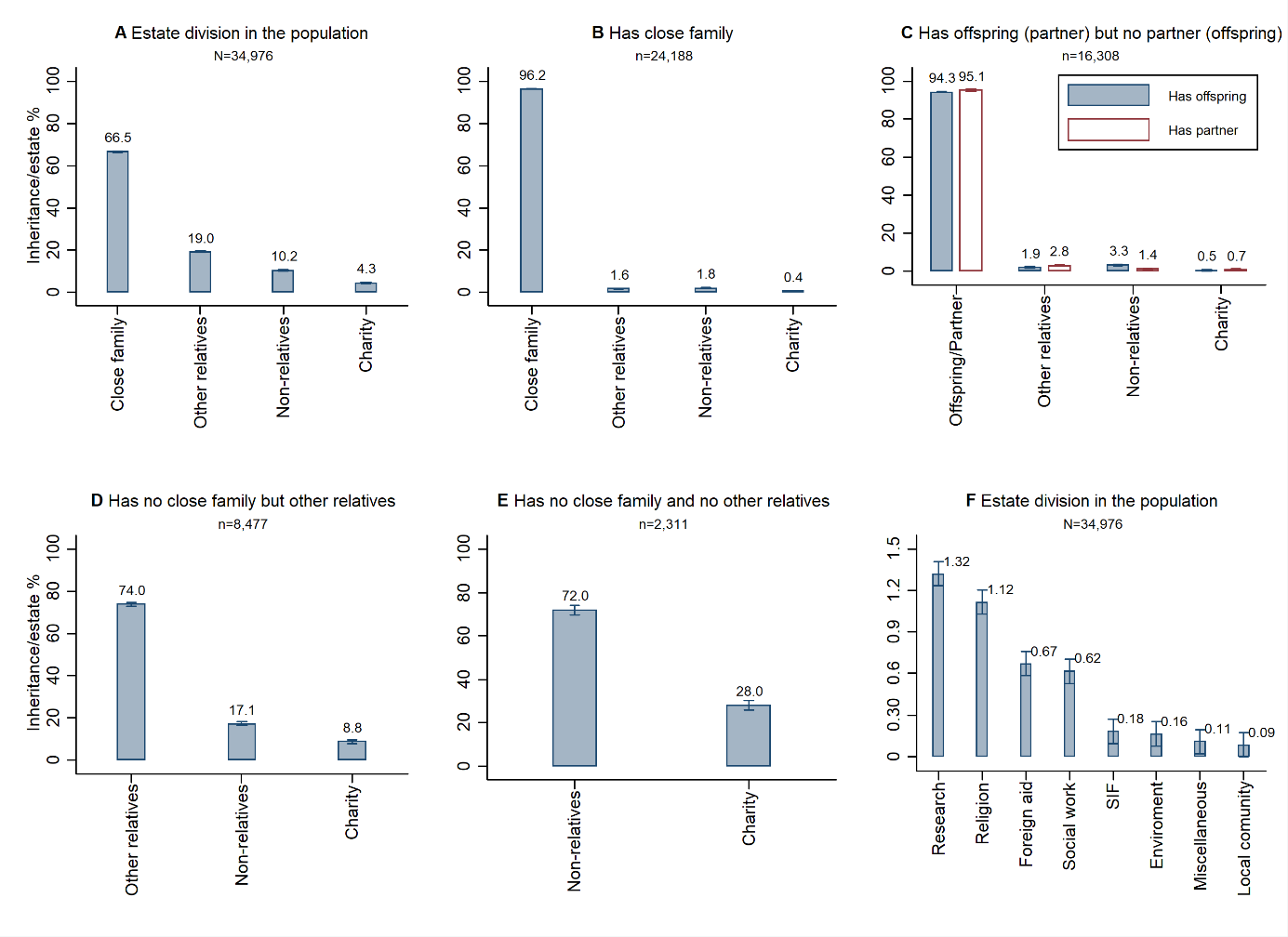


**Figure S2. Estate divisions for decedents with testaments.** For details regarding the estimations, see section II in the main text. The bars are accompanied by 99% confidence intervals.

# S3 Swedish Inheritance Legislation

Swedish inheritance laws stipulate how the assets of a deceased individual should be distributed among those who are entitled to an inheritance from the decedent, either by the succession order (legal heirs) or through a testament (legatees).

The succession order is based on the level of relatedness between the decedent and the heir. Closer relatives inherit before more distant relatives. The decedent’s relatives are classified into three categories of legal heirs. Each category has to be empty of heirs before heirs in the next category are considered.

Moreover, it follows from the law that if the heir is deceased, his or her offspring become legal heirs and entitled to the inheritance. The default rule is that the estate is divided into equal shares between the legal heirs within the given category.

The first category of legal heirs contains the decedent’s offspring (i.e. children, grandchildren and so on). The heirs in this category are referred to as direct heirs and the children (biological and adopted) are the first in line to inherit.

The second category contains the decedent’s parents, siblings and sibling’s offspring (i.e. nephews and nieces). The relatives in this category inherit the estate, in the stated order, if there are no heirs in the first category.

The third category contains the decedent’s grandparents and their children (i.e. aunts and uncles). First cousins and more distant relatives are not legal heirs.

Besides the categories of legal heirs, there are specific rules concerning surviving spouses. The default rule is that the surviving spouse inherits the entire estate of the late spouse with full ownership. However, if the decedent has legal heirs, the spouse does not inherit with full ownership but rather with the right of free disposal. This means that the spouse is free to spend the inheritance from the late spouse but prohibited from bequeathing it. The inheritance right of the legal heirs is postponed until the surviving spouse eventually passes away.

Moreover, the inheritance right of a surviving spouse is limited if the decedent had children that are not common with the surviving spouse (e.g. from a previous marriage). These children are entitled to the inheritance immediately upon their parent passing away.

If there is no spouse or no legal heirs in any of the three groups, the estate goes to a public fund, The Swedish Inheritance Fund (SIF). The purpose of SIF is to support non-profit activities aiming at supporting children, youth as well as people with disabilities. In this sense, SIF operates much like an ordinary charity.

Unlike married spouses, cohabitants do not inherit each other by default. That is irrespective of whether or not they have common children. The common children are entitled to the inheritance at the death of the first parent if the parents cohabitated.

The succession order can be set aside by a testament. A testament is a legally binding document stipulating the last will of the deceased concerning who should inherit the estate (e.g. a cohabitant or a charity) or how particular assets should be distributed. For a testament to be valid, it has to be signed by the testator and confirmed, through signature, by at least two witnesses. While it is required that the witnesses understand that they confirm a testament, they may not be allowed to read the content. A testament is revocable at any time prior to death, either by tearing it up or by replacing it with a new one.

The inheritance right of direct heirs (i.e. offspring) is protected by the rule of statutory portion. This rule implies that a direct heir is entitled to a minimum of 50 percent of what he or she would have received in the absence of the testament (i.e. the statutory portion). Thus, an individual with offspring can bequeath at most half of his or her property to others than his or her offspring.

# S4 Data on estate divisions

Our main data source is a population-wide register called Belinda, which is held by Statistics Sweden. The register originates from the Swedish Tax Agency and contains detailed accounts of the estates of, and inheritances from, all individuals who passed away in Sweden during the period of 2002–2004 and all of their biological and non-biological heirs. Data are available from this period as the Tax Agency was obliged to electronically codify all estate reports starting in July 2001; however, this obligation was suspended in 2005 when the inheritance tax was completely abolished.

Information about the decedents includes the value of net worth at death and its main components (total assets and total debts), the value of the estate, a list of the heirs and beneficiaries of testaments, special rules that apply to the estate and the bequests (e.g. testament, marital agreement and life insurance policy) and personal details (e.g. identity number, marital status and date of death).

Information about heirs and beneficiaries of testaments includes the value of their received inheritance from the deceased (including those of zero value) and personal details (e.g. identity number and relationship to the decedent).

The point of departure for our analyses is the population of decedents who passed away during the two years 2002 and 2003. The reason for not considering those who passed away in 2004 is that the inheritance tax on spousal bequests was repealed at the beginning of 2004. Bequests to charitable organizations were exempted from taxation throughout the period of 2002–2004. The tax repeal changed the relative price of giving to individuals, and to rule out the possibility that changed tax incentives confound the empirical estimates, we exclude decedents and heirs following the tax repeal.

187,827 individuals passed away during the period of 2002–2003. For 186,020 decedents, we are able to identify any heir or beneficiary of will (person or organization), in total 588,031 heirs. From this initial population, we exclude some decedents (and, indirectly, their heirs as well). Table S4 shows how many decedents (and heirs) are excluded due to each criterion. Note that a decedent may be excluded due to more than one of these criteria. In addition, the table reports the total amount of estate wealth affected by each criterion.

First, we exclude individuals for whom we have indications that the inheritances are misreported. Some heirs are reported as having inherited zero wealth although the deceased donor passed away with a positive estate. For 73 percent of these cases, we can calculate the correct value of the inheritance by following the succession law. For the remaining cases, we are unable to determine the estate distribution (e.g. because the decedent has a testament) and, subsequently, the correct value of the inheritance, which is why these cases are excluded from the analyses.

Second, we exclude decedents for whom there is at least one heir with missing information concerning his or her relationship with the decedent. This is because we cannot classify the heir as belonging to a specific heir group if we do not know the heir’s relationship to the decedent.

Third, we exclude decedents for whom personal identity number (PIN) is missing. This is because we cannot link information from other registers if the personal identity number is missing.

Fourth, we exclude decedents who are younger than 18 years old. This is because individuals younger than 18 are not allowed to freely testate their wealth.

The exclusion criteria leave us with 176,279 decedents. However, for the analysis we restrict the focus to the population of decedents with estates of positive values since the outcome of interest is the share of the estate transferred to different groups of heirs. Excluding decedents with estates of zero value (referred to as exclusion criterion 5) leaves us with a study population of 146,657 decedents and 460,034 heirs. In Table S2.6, we report descriptive statistics for decedents with estates of zero value and it can be seen that they on average are younger, have lower education and income, and are males to a larger extent than the decedents in the study population.

| Table S4: Exclusion criteria, study population and analysis sample | | | | |
| --- | --- | --- | --- | --- |
|  |  | No. of decedents | No. of heirs | Total estate wealth |
| Initial population: | | 186,020 | 588,031 | 50,140,397,844 |
|  | |  |  |  |
| Exclusion criteria: | |  |  |  |
| 1 | Missing data on inheritances | 8,919 | 41,100 | 3,322,455,769 |
| 2 | Missing data on relationship | 463 | 3,625 | 257,891,023 |
| 3 | Missing data on PIN | 256 | 1,078 | 368,303,317 |
| 4 | Younger than 18 years | 372 | 770 | 17,044,619 |
|  |  |  |  |  |
|  | Fulfills any of (1)-(4) | 9,741 | 45,395 | 3,594,947,029 |
|  | After exclusion criteria (1)-(4) | 176,279 | 542,636 | 46,545,450,815 |
| 5 | Zero estate | 29,622 | 82,602 | 0 |
| Study population | | 146,657 | 460,034 | 46,545,450,815 |

# S5 Grouping of recipients of inheritances

The Belinda database contains a variable denoting the heir’s relationship with the decedent. All individuals, who are heirs according to the succession rule or beneficiaries of testaments, are listed in the estate report. Surviving cohabitants should also be listed in the estate report, even though cohabitants are not legal heirs according to the inheritance law. It is important to note that the heirs and beneficiaries of testaments should be listed even if they do not receive anything. For example, a decedent may have siblings as the only living relatives. The siblings are heirs according to the succession rule. However, unlike offspring, siblings are not entitled to a statutory portion, so if the decedent has written a testament stipulating that the entire estate should go to a charity, the siblings inherit nothing but are nevertheless listed in the estate report (and our data) together with the charity. In Table S5.1, we list the heir-to-decedent relationships present in the data.

| Table S5.1: Heir-decedent relationships in analysis sample | | |
| --- | --- | --- |
| Relationship | Number | Percent |
| **Close family** | **304,096** | **66.04** |
| Child | 225,796 | 49.08 |
| Grandchild | 21,584 | 4.69 |
| Great-grandchild and its offspring | 1,083 | 0.24 |
| Spouse | 52,230 | 11.35 |
| Registered partner | 7 | <0.001 |
| Cohabitant | 3,396 | 0.74 |
| **Other relatives** | **128,080** | **27.84** |
| Mother | 2,007 | 0.44 |
| Father | 1,339 | 0.29 |
| Sibling | 34,510 | 7.50 |
| Sibling’s child | 71,886 | 15.63 |
| Sibling’s grandchild and its offspring | 17,915 | 3.90 |
| Paternal grandmother | 1 | <0.001 |
| Maternal aunt | 179 | 0.04 |
| Maternal uncle | 103 | 0.02 |
| Paternal aunt | 84 | 0.02 |
| Paternal uncle | 56 | 0.01 |
| **Non-relatives** | **20,501** | **4.46** |
| Friends and acquaintances | 18,380 | 4.00 |
| Child’s partner | 245 | 0.05 |
| Foster child | 571 | 0.12 |
| Stepchild | 1,305 | 0.28 |
| **Charity** | **7,357** | **1.60** |
| **Total** | **460,034** | **100** |

For the analysis, we group the recipients into six groups (four major and two sub-groups).

*Offspring*: Sub-group containing the decedent’s offspring: children, grandchildren, great-grandchildren, etc.

*Partner*: Sub-group containing surviving spouse, registered partner and cohabitant.

*Close family:* Group that is the union of the sub-groups *Offspring* and *Partner.*

*Other relatives*: Group containing relatives other than offspring or partner: mother, father, siblings, siblings’ offspring, grandmothers, grandfathers, uncles or aunts.

*Non-relatives*: Group containing non-related (physical) individuals. Most individuals in this group are listed as having no relationship with the decedent. They are likely to consist of the decedent’s friends and other acquaintances and possibly also some distant relatives (who do not inherit according to the succession order; for example, first cousins). In this group, we include also a few heirs listed as stepchildren, foster children or child’s spouse or partner.

*Charity:* The Belinda database includes information on organizations receiving inheritances. These are identified with an organization number and/or name. Researchers are not allowed to conduct analyses using the names of the organizations. However, Statistics Sweden has provided us with a complete list of the names of the organizations and allowed us to group them by cause. We have classified each organization by its main cause, as stated by its webpage or registration at the Tax Agency. For example, the Swedish Cancer Society is a non-profit organization with the purpose of raising money for cancer research, which is why we classify it as a cancer research charity. The list not only contains regular charities but also government institutions (e.g. universities, municipalities), religious organizations (e.g. churches and aid organizations), environmental organizations (e.g. WWF), organizations working for the local community and animal rights organizations. However, from the perspective of the donor, it is reasonable to assume that bequests to all of these organizations are charitable in a wider sense. Our classification results in 22 categories of charitable organizations (including the Swedish Inheritance Fund). Table S5.2 details the categories and their incidence in data. When referring to the group *Charity* in the analyses, we refer to the aggregate group containing these 22 categories.

| Table S5.2: Groups and categories of charitable organizations | | |
| --- | --- | --- |
| Charity | Number | Percent |
| **Swedish Inheritance Fund** | **1,905** | **25.89** |
| **Religion** | **1,773** | **24.10** |
| **Research** | **1,348** | **18.32** |
| Cancer | 777 | 10.56 |
| Heart and lung | 191 | 2.60 |
| Health general | 262 | 3.56 |
| Brain | 58 | 0.79 |
| Education | 51 | 0.69 |
| Research general | 9 | 0.12 |
| **Foreign aid** | **1,043** | **14.18** |
| **Social work** | **748** | **10.17** |
| Social work | 389 | 5.29 |
| Disability | 183 | 2.49 |
| Care | 147 | 2.00 |
| Abuse | 29 | 0.39 |
| **Environment** | **199** | **2.70** |
| Animals | 152 | 2.07 |
| Environment | 47 | 0.64 |
| **Miscellaneous** | **194** | **2.64** |
| Association general | 137 | 1.86 |
| Unknown cause | 28 | 0.38 |
| Culture | 27 | 0.37 |
| Politics | 2 | 0.03 |
| **Local community** | **147** | **2.00** |
| Local culture | 96 | 1.30 |
| Sports | 46 | 0.63 |
| Municipality | 5 | 0.07 |
| **Total** | 7,357 | 100 |

# S6 Construction of analysis samples

The samples are summarized in Table S6.

*Population*: Contains all decedents with a positive estate, in total 146,657 individuals. Not all decedents have a full choice set of recipient groups, in particular those with no close family members.

*Has close family*: Contains decedents with at least one close family member (offspring and/or a partner), in total 122,718 individuals. These decedents are assumed to have a full choice set of recipient groups.

*Has offspring but no partner*: Contains decedents with offspring but no partner, in total 67,093 individuals.

*Has partner but no offspring*: Contains decedents with partner but no offspring, in total 19,931 individuals.

*Has no close family but other relatives*: Contains decedents with no close family but other relatives, in total 20,804individuals.

*Has no close family and no other relatives*: Contains decedents with no close family and no other relatives, in total 3,135 individuals.

| Table S6: Analysis samples | | | | | | | | |  |  |
| --- | --- | --- | --- | --- | --- | --- | --- | --- | --- | --- |
| Group of heirs: | | Close family | | Other relatives | Non-relatives | Charities | No. of decedents | No. of recipients | Used in Fig. 1, panel: | |
|  | | Offspring | Partner |  |  |  |  |  |  | |
| Types of individuals in group: | | Children, grandchildren, great-grandchildren | Spouse, registered partner, cohabitant | Parents, siblings, siblings’ offspring grandparents, uncles and aunts | Friends and acquaintances,  stepchildren, foster children, child’s spouse | Swedish Inheritance Fund, research,  religion, foreign aid, social work, environment and animals, local community, miscellaneous |  |  |  | |
| Population | | X | X | X | A | A | 146,657 | 460,034 | A | |
| Has close family | | R | | X | A | A | 122,718 | 329,468 | B | |
| Has offspring but no partner | | R |  | X | A | A | 67,093 | 168,531 | C | |
| Has partner but no offspring | |  | R | X | A | A | 19,931 | 38,725 | C | |
| Has no close family but other relatives | |  |  | R | A | A | 20,804 | 120,973 | D | |
| Has no close family and no other relatives | |  |  |  | A | A | 3,135 | 9,593 | E | |
| Note: Potentially in choice set (X), Required to be in choice set (R), Excluded from choice set (Empty), Always in the choice set (A) | | | | | | | | | | |

# S7 Measurement of estates and inheritances

The Belinda database contains information on the value of the decedents’ estates (i.e. the amount transferred to the recipients). We take this amount as given in the register and adjust it to the price level of 2003.

Inheritances are given in pre-tax values but net of any cedes. Ceding was a way for heirs to reduce the tax payment by passing the entire inheritance or a part of it to their children. There is information concerning the ceded amounts in the data and we define the inheritance as the sum of the inheritance amount (as listed in the data) and the ceded amount and adjust it to the price level of 2003. Some recipients receive two inheritances when the decedent passes away, typically a child who receives one inheritance from the recently deceased parent and one from a previously deceased parent. For these cases, we only consider the most recent inheritance (i.e. the one from the decedent we observe in the data).

The main outcome variable in the analysis is a variable measuring how much of the estate is transferred to each respective group of heirs. We calculate this variable as follows. First, we calculate the sum of inheritances going to each group. Second, we divide the inheritance sum by the value of the estate. For example, for a decedent with an estate of SEK 1 million and two children (and no surviving partner), each receiving SEK 400,000, and a charity receiving SEK 200,000, the share going to close family is 0.8 and the share going to charity is 0.2. The shares going to the other groups are, in this case, zero.

# S8 Descriptive statistics for the decedents

In this section, we provide descriptive statistics for the decedents’ characteristics in the analysis samples, reported in Table S8.1. The characteristics we consider are: estate value (in SEK), age (in years at year of demise), gender (indicator for female (=1)), number of heirs, highest level of education (indicators for primary or lower secondary (=1), upper secondary (=1), university (=1), missing (=1)), total income (sum of taxable labor and capital incomes, in SEK). Level of education and income are measured in the year prior to the year of the demise. Many decedents were educated before the education registers were created, why this variable is missing for a large share of decedents. Information on the estate value and number of heirs is obtained from the Belinda database, while information on age, gender and level of education is obtained from the Income and Tax Register, which is a population-wide register from the Tax Agency. We report sample means and, for continuous variables, standard deviation (in parentheses). The means for indicator variables are reported in percent.

In Table S8.2, we report descriptive statistics for decedents with estates of zero value. The statistics are the same ones as in Table S2.5, described above.

In Table S8.3, we report descriptive statistics for decedents with close family and who have bequeathed to charity.

| Table S8.1: Descriptive statistics for the decedents | | | | | | | | | | |
| --- | --- | --- | --- | --- | --- | --- | --- | --- | --- | --- |
|  | Population | | Has close family | | Has no close family but other relatives | | Has no close family and no other relatives | | |  |
|  | All | Testament | All | Testament | All | Testament | | All | Testament | |
|  | (1) | (2) | (3) | (4) | (5) | (6) | | (7) | (8) | |
| Age (years) | 80.5 | 81.9 | 80.6 | 80.3 | 79.2 | 85.3 | | 83.7 | 85.0 | |
|  | (11.7) | (10.3) | (10.9) | (10.5) | (15.7) | (9.2) | | (9.4) | (8.7) | |
| Female (%) | 51.9 | 51.3 | 51.6 | 45.7 | 52.4 | 64.4 | | 58.1 | 61.3 | |
| Level of education (%) |  |  |  |  |  |  | |  |  | |
| Primary or lower secondary | 12.2 | 9.3 | 12.0 | 10.7 | 13.8 | 6.1 | | 8.8 | 6.4 | |
| Upper secondary | 9.1 | 8.0 | 9.3 | 9.8 | 8.5 | 3.9 | | 5.2 | 4.0 | |
| University | 3.8 | 4.4 | 3.9 | 5.5 | 3.2 | 1.7 | | 1.9 | 1.8 | |
| Missing | 75.0 | 78.4 | 74.8 | 74.0 | 74.6 | 88.3 | | 84.1 | 87.8 | |
| Total income (SEK) | 151,455 | 180,470 | 153,922 | 195,186 | 137,233 | 145,751 | | 149,254 | 154,003 | |
|  | (233,395) | (428,342) | (238,594) | (480,045) | (205,387) | (292,839) | | (195,972) | (209,007) | |
| Estate (SEK) | 317,376 | 517,783 | 292,974 | 517,618 | 422,920 | 505,595 | | 572,191 | 564,210 | |
|  | (1,455,589) | (2,848,232) | (1,530,953) | (3,333,959) | (880,091) | (1,102,536) | | (1,437,699) | (1,407,643) | |
| Number of heirs | 3.1 | 4.1 | 2.7 | 3.1 | 5.8 | 6.8 | | 3.1 | 3.5 | |
|  | (2.9) | (4.0) | (1.9) | (2.6) | (5.4) | (5.9) | | (3.4) | (3.6) | |
| *N* | 146,657 | 34,976 | 122,718 | 24,188 | 20,804 | 8,477 | | 3,135 | 2,311 | |
| Note: All values represent sample means (standard deviations in parentheses). All variables except age and number of heirs are measured the year prior to the demise. Total income and estate are reported in SEK in the price level of 2003. The exchange rates as of December 30, 2003: 7.8 SEK/USD and 9.0 SEK/EUR. | | | | | | | | | | |

| Table S8.2: Descriptive statistics for the decedents with estates of zero value | |
| --- | --- |
| Age (years) | 71.6 |
|  | (17.5) |
| Female (%) | 48.9 |
| Level of education (%) |  |
| Primary or lower secondary | 23.4 |
| Upper secondary | 19.2 |
| University | 5.5 |
| Missing | 51.9 |
| Total income (SEK) | 120,938 |
|  | (108,640) |
| Estate (SEK) | 0 |
| Number of heirs | 2.8 |
|  | (1.9) |
| *N* | 29,773 |
| All values represent sample means (standard deviations in parentheses). All variables except age and number of heirs are measured the year prior to the demise. Total income and estate are reported in SEK in the price level of 2003. The exchange rates as of December 30, 2003: 7.8 SEK/USD and 9.0 SEK/EUR. | |

| Table S8.3: Descriptive statistics for the decedents with close family and who have bequeathed to charity | |
| --- | --- |
| Age (years) | 81.8 |
|  | (9.0) |
| Female (%) | 44.9 |
| Level of education (%) |  |
| Primary or lower secondary | 10.0 |
| Upper secondary | 5.5 |
| University | 5.5 |
| Missing | 79.1 |
| Total income (SEK) | 252,083 |
|  | (1,501,970) |
| Estate (SEK) | 1,066,951 |
|  | (5,211,402) |
| Number of heirs | 5.8 |
|  | (4.8) |
| *N* | 788 |
| All values represent sample means (standard deviations in parentheses). All variables except age and number of heirs are measured the year prior to the demise. Total income and estate are reported in SEK in the price level of 2003. The exchange rates as of December 30, 2003: 7.8 SEK/USD and 9.0 SEK/EUR. | |

# S9 Data access

The main analyses of the paper are based on data from the Belinda database and the Income and Tax Register, which are held and maintained by Statistics Sweden (SCB). We are not allowed to directly share the data according to our agreement with SCB.  The data can be accessed by others in the same manner by which the authors obtained them. Data access requests should be sent to SCB (mikrodata.individ@scb.se).

In addition to these data sources, we use survey data from NOVUS; see section II in main text and S1. The NOVUS data is available as a separate supporting information file to this article.

# S10 Details on the distribution of bequests to charities

In this section, we report the details for the analysis of the distribution of estate wealth to the different groups of charities. As noted in S5, there are in total 22 charity categories in our data. For illustrational purposes, we divide them into 8 well-defined groups of beneficiaries and one residual group, described below.

*Swedish Inheritance Fund* (*SIF*): See description in S3.

*Research*: Group containing organizations with the purpose of raising money for research. Examples of organizations include medical research charities (e.g. The Swedish Cancer Society and the Swedish Heart-Lung Foundation), research institutes and universities.

*Religion:* Group containing religious communities such as churches and congregations. It is possible to stipulate in a testament that a part of the estate or a specific amount should be transferred to the cemetery administration (governed by the Church of Sweden) and be used to cover expenses associated with the future maintenance of the grave, a so-called grave care legacy. We consider this a form of charitable bequest to the Church of Sweden. In reality, only 0.49% of the decedents stipulate a grave care legacy in the testament and the share of total estate wealth going to grave care legacies is negligible (<0.01%).

*Foreign aid:* Group containing organizations with the purpose of raising money to people in need. Examples of organizations include The International Red Cross and Médecins Sans Frontièrs. Common for these organizations is that the eventual recipients of the inheritances are poor or unfortunate people in countries other than Sweden.

*Social work:* Group containing organizations with the purpose of raising money for or directly helping unfortunate people in Sweden, such as disabled, homeless and poor individuals. Examples of organizations in this category include the Salvation Army, homeless shelters, Alcoholics Anonymous and organizations working for better living conditions for people with disabilities. This group also contains nursing and retirement homes.

*Environment*: Group containing organizations working for the protection of the environment (e.g. preservation of the wilderness and endangered species). Examples of organizations in this group include the World Wildlife Fund (WWF) and Greenpeace. The group also contains organizations working for animal rights and organizations helping abused or abandoned pets (e.g. cat shelters).

*Local community*: Group containing organizations connected to the local community. Organizations in this group include municipalities (administrative division), sports clubs and local history societies. Common for these organizations is that the eventual recipients of the inheritances belong to a local geographic area.

*Miscellaneous*: Group containing organizations that do not fit elsewhere and which receive too small amounts, in aggregate terms, to constitute independent groups. Organizations in this category include political parties, cultural institutions (e.g. museums), general associations and clubs working primarily for the members (e.g. Rotary, Freemasonry) and organizations we are unable to classify since we have no information on their cause. It should be noted that trust funds are extremely rare in Sweden, and even more so in the early 2000s. This is not surprising given that trust funds were liable to inheritance taxation. From the names of the funds in our data, the size of the inheritances they receive and the taxes they pay, we are confident that we have a negligible amount of inheritances transferred to trust funds in our data.

Figure S10 provides estimates of how the share of the estate transferred to *Charity* in Figure 1 is distributed among the groups of charities. The estimates are obtained from a regression of model (2) (see section II), but with the group indicators for *cf*, *or*, *nr* and *ch* changed to indicators for the charity groups described above.

From Figure S10, we see that the largest share goes to the *Swedish Inheritance Fund* (*SIF*) (0.42%). This is not surprising since it is the default recipient of bequests from decedents who pass away without legal heirs. The second largest share goes to *Research* (0.34%) and the third largest share goes to *Religion* (0.28%). The shares going to *Foreign aid* and *Social work* are both 0.17% and the one to *Environment* is 0.04% of the estate. 0.03% and 0.02% of the estate goes to the groups *Miscellaneous* and *Local community* respectively.

**
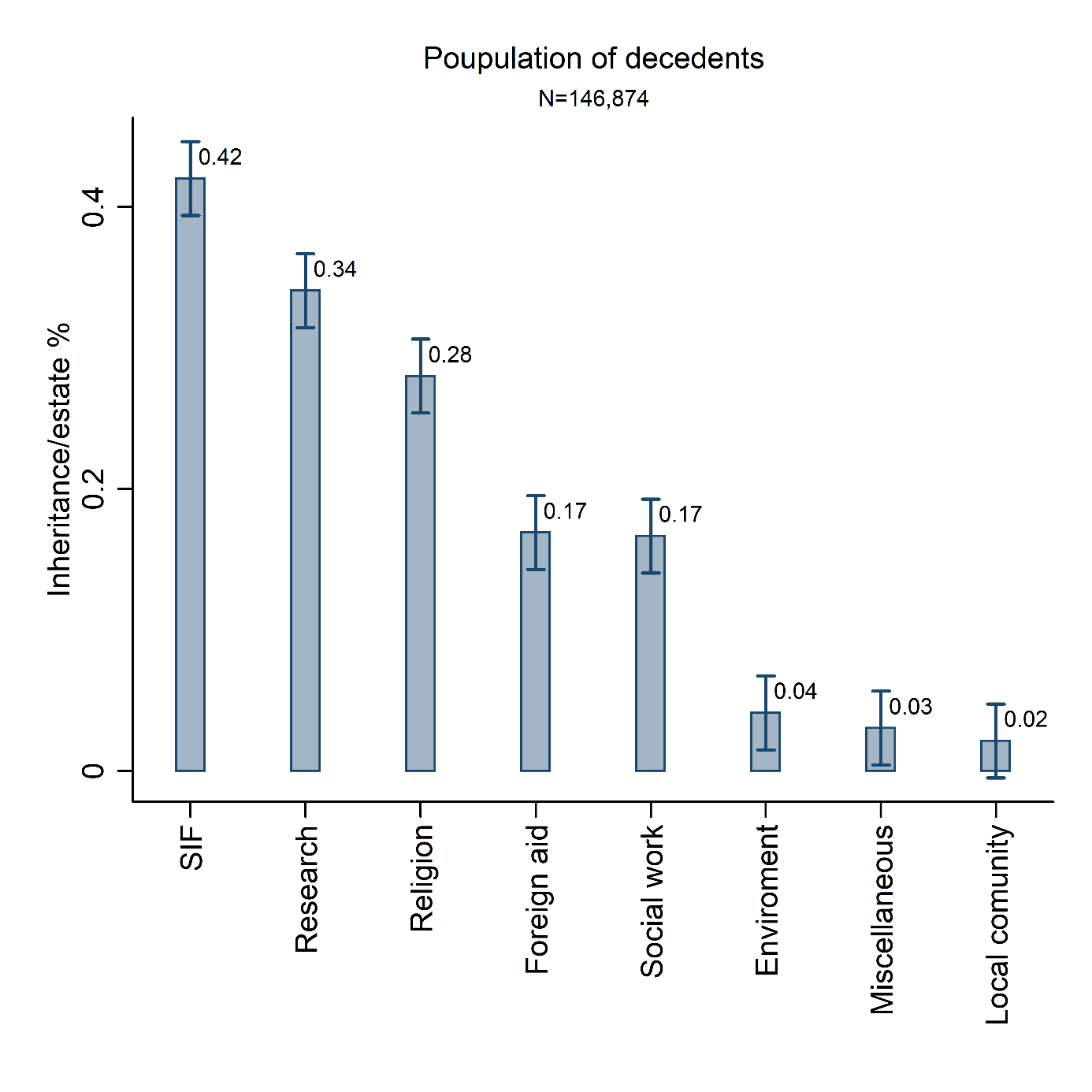
**

**Figure S10. Bequests to different groups of charities.** For details regarding the estimations, see section II in the main text. The bars are accompanied by 99% confidence intervals.

# S11 Detailed estimation results.

| Table S3.1: Detailed results for figures 1-5. | | | | | | |
| --- | --- | --- | --- | --- | --- | --- |
|  | Figure 1. | Figure 2. | Figure 3. | Figure 4. | Figure 5 | |
| Sample | All | Has close family | Has no close family but other relatives | Has no close family and no other relatives | Has offspring but no partner | Has partner but no offspring |
|  | (1) | (2) | (5) | (6) | (3) | (4) |
| Close family | 0.828*** | 0.990*** |  |  |  |  |
|  | (0.000698) | (0.000176) |  |  |  |  |
| Other relatives | 0.130*** | 0.00559*** | 0.884*** |  | 0.00366*** | 0.0217*** |
|  | (0.000698) | (0.000176) | (0.00167) |  | (0.000218) | (0.000779) |
| Non-relatives | 0.0271*** | 0.00397*** | 0.0765*** | 0.606*** | 0.00552*** | 0.00438*** |
|  | (0.000698) | (0.000176) | (0.00167) | (0.00840) | (0.000218) | (0.000779) |
| Charity | 0.0147*** | 0.000882*** | 0.0392*** | 0.394*** | 0.000848*** | 0.00239*** |
|  | (0.000698) | (0.000176) | (0.00167) | (0.00840) | (0.000218) | (0.000779) |
| Offspring |  |  |  |  | 0.990*** |  |
|  |  |  |  |  | (0.000218) |  |
| Partner |  |  |  |  |  | 0.972*** |
|  |  |  |  |  |  | (0.000779) |
| N | 146,657 | 122,718 | 20,804 | 3,135 | 67,093 | 19,931 |
| For details regarding the estimations, see section II in the main text. Note. Standard errors in parentheses  *** p<0.01, ** p<0.05, * p<0.1 | | | | | | |
